# Mechanisms of ferroptosis in nonalcoholic fatty liver disease and therapeutic effects of traditional Chinese medicine: a review

**DOI:** 10.3389/fmed.2024.1356225

**Published:** 2024-03-25

**Authors:** Nan Wang, Hanyun Que, Qiulin Luo, Wenxin Zheng, Hong Li, Qin Wang, Jian Gu

**Affiliations:** ^1^College of Pharmacy, Southwest Minzu University, Chengdu, China; ^2^BMI Center for Biomass Materials and Nanointerfaces, College of Biomass Science and Engineering, Sichuan University, Chengdu, China

**Keywords:** ferroptosis, nonalcoholic fatty liver disease, traditional Chinese medicine, nonalcoholic steatohepatitis, mechanisms

## Abstract

Nonalcoholic fatty liver disease (NAFLD) is characterized by excessive accumulation of fat in hepatocytes (nonalcoholic fatty liver (NAFL)), and lobular inflammation and hepatocyte damage (which characterize nonalcoholic steatohepatitis (NASH) are found in most patients). A subset of patients will gradually develop liver fibrosis, cirrhosis, and eventually hepatocellular carcinoma, which is a deadly disease that threatens human life worldwide. Ferroptosis, a novel nonapoptotic form of programmed cell death (PCD) characterized by iron-dependent accumulation of reactive oxygen radicals and lipid peroxides, is closely related to NAFLD. Traditional Chinese medicine (TCM) has unique advantages in the prevention and treatment of NAFLD due to its multicomponent, multipathway and multitarget characteristics. In this review, we discuss the effect of TCM on NAFLD by regulating ferroptosis, in order to provide reference for the further development and application of therapeutic drugs to treat NAFLD.

## 1 Introduction

Nonalcoholic fatty liver disease (NAFLD) is the leading cause of prolonged liver diseases (1). Due to its high frequency, difficult diagnosis, and complicated pathophysiology, NAFLD has become a major challenge (2). NAFLD is defined as 5% fat accumulation in hepatocytes without excessive alcohol usage and other secondary variables of fatty liver degeneration such as obesity, type 2 diabetes or extrametabolic syndromes. NAFLD is characterized by hepatocyte loss, inflammatory infiltration, and fibrosis and can start as simple steatosis and progress to nonalcoholic steatohepatitis (NASH) with or without fibrosis of the liver (3). Lipid accumulation in the liver can increase reactive oxygen species levels, increase cell apoptosis, and accelerate ageing. Increases in lipids, oxidative damage, stress in the endoplasmic reticulum, and NAFLD development are correlated with lipotoxicity (4). Severe NAFLD and NASH may advance to conditions such as cirrhosis and hepatocellular cancer, eventually leading to organ failure (5).

Compared with apoptosis, ferroptosis has recently been identified as a novel type of programmed cell death. In 2003, Dixon et al. found a nonapoptotic form of tumor death in a study on erastin, a small-molecule chemical medication with antitumor effects. Based on its regulatory property, Dixon et al. called this type of cell death ferroptosis in 2012 (6). Ferroptosis is an aberrant type of metabolism mediated by iron ions and the disruption of intracellular lipid oxide metabolism. The accumulation of lipid reactive oxygen species (reactive oxygen species (ROS) occurring during lipid peroxidation) and intracellular redox imbalance that results from an impaired glutathione (GSH)-dependent lipid peroxide repair pathway reduces the antioxidant capacity of cells. (7). Several investigations have revealed that ferroptosis significantly contributes to the aetiology of NAFLD. Additionally, high iron levels may exacerbate NAFLD by causing liver cell enlargement, inflammation, and fibrosis, which can cause NAFLD to develop into NASH. As a result, inhibiting ferroptosis may be a potential treatment approach for NAFLD. (8). Multiple investigations have revealed that traditional Chinese medicine (TCM) monomers and compounds have regulatory effects on ferroptosis. Moreover, TCM has exceptional benefits for treating metabolic illnesses, and because of its strong therapeutic efficacy and few adverse effects, it offers a wide range of potential applications in the treatment therapy of NAFLD (9).

In this review, we describe the machinery controlling ferroptosis and summarize the role of ferroptosis in the development of NAFLD. In addition, we discuss the research progress on TCM monomers and compounds that target ferroptosis in the treatment of NAFLD, as well as existing issues and potential future study areas. This will offer a theoretical foundation and research plan for TCM treatment of NAFLD.

## 2 Ferroptosis

### 2.1 Ferroptosis characteristics

Ferroptosis causes cell membrane rupture and blistering, mitochondrial shrinkage, mitochondrial ridge decrease or removal, increased membrane density, and normal nuclear morphology, but there is no chromatin condensation, according to the ultrastructure of the cells. Under an electron microscope, it is also possible to see that intracellular mitochondria are shrunken and membrane density is increased (10). These morphological characteristics aid in the differentiation of ferroptosis from apoptosis, necrosis, and autophagy. Ferroptosis is characterized by the accumulation of radical oxygen and iron ions, stimulation of the mitogen-activated protein kinase (MAPK) pathway, a reduction in cystine absorption, and glutathione depletion (11). In addition, erastin or RSL3, which are classical ferroptosis activators, can cause abundant intracellular iron ion accumulation and thus inhibit the intracellular antioxidant system and then increase damage caused by oxidation by generating ROS through the Fenton reaction. Fatal lipid peroxidation, which is one of the primary causes of ferroptosis, can be inhibited by many by a variety of synthetic antioxidants, such as ferritin-1 and rilpastatin-1, can be accelerated by a compromised antioxidant system (12).

In addition, the expression of several genes or proteins can be used to indicate ferroptosis. For example, Prostaglandin Endoperoxide Synthase 2 (PTGS2/COX-2), which is a prostaglandin endoperoxide synthase and a key enzyme in prostaglandin biosynthesis, acts as an important marker of ferroptosis in ischaemic stroke (13). By affecting the expression of the ferroptosis signalling molecules glutathione peroxidase 4 (GPX4), solute carrier family 7 member 11 (SLC7A11), PTGS2 and ATP5G3, resveratrol glycosides can play a therapeutic role in the treatment of traumatic brain injury (14). In addition, ten genes (such as anti-ACSL3 antibody (ACSL3), GPX4, and SLC7A11) associated with ferroptosis have been postulated to have regulatory roles in lung adenocarcinoma (15). GPX4 and SLC7A11 are also important in hepatocellular carcinoma (HCC). High levels of ferroptosis can be found in radioresistant HCC, which is involved in the radiosensitization of liver cancer. Cytokine signalling 2 (SOCS2), which is a predictor of the prognosis of liver cancer after radiotherapy, promotes the ubiquitination and degradation of SLC7A11 and promotes ferroptosis (16). The lncRNA HEPFAL can promote ferroptosis by reducing the expression of solute carrier family 7 member 11 (SLC7A11) and increasing the levels of lipid ROS and iron (two surrogate markers of ferroptosis) (17). However, the exact mechanism by which ferroptosis is associated with these diseases is unclear and more research is needed.

### 2.2 Ferroptosis metabolic mechanism

#### 2.2.1 Lipid metabolism

Monounsaturated fatty acids, polyunsaturated fatty acids, and saturated fatty acids (that lack double bonds) are the three types of fatty acids. Lipid peroxidation caused by free radicals mostly affects unsaturated fatty acids in cell membranes (18). Studies have shown that the most important sources of these unsaturated fatty acids include arachidonic acid (AA) and linous acid, which are widely present in cells. Evidence also confirmed that the addition of these two unsaturated fatty acids to cell cultures can accelerate the occurrence of ferroptosis. The specific oxidation of arachidonic acid (AA) is mainly regulated by three enzymes AA is activated to AA-CoA by acyl Acyl CoA synthetase long chain protein 4 (ACSL4), the activated lipid molecules are esterified with phosphatidylcholine to form AA-PE by lysophosphatidylcholine acyltransferase 3 (LPCAT3), and lipid peroxidation occurs via the catalysis of lipoxygenase protein family (LOXs). These enzymes all play important roles in ferroptosis; ACSL4 is an important indicator protein in ferroptosis, and ALOX5 and ALOX12 in the LOX enzyme family are also targets of many ferroptosis inducers. Iron increases the activity of arachidonate lipoxygenase (ALOX), which is crucial for lipid peroxidation and oxygen homeostasis. This is especially true for the peroxidation of unsaturated fatty acids in phospholipids in the context of ferroptosis. (10). The iron-dependent build-up of potentially hazardous reactive oxygen species in lipids is what defines ferroptosis; hence, lipid metabolism plays a key role in the initiation and execution of cell death. When glutathione is available, a functional selenoprotein known as GPX4 transforms lipid peroxides to nontoxic lipid alcohols. Large amounts of lipid peroxides build up in the cell membrane, mitochondrial membrane, or endoplasmic reticulum membrane when the activity of this protein is compromised in the presence of iron (19). Since these lipid ROS cannot be reduced, reactive oxygen species in lipids build up over time, which kills the cells.

Ferroptosis cannot occur without specific lipid oxidation and natural antioxidant mechanisms must be impaired. During normal cellular metabolism, lipids and oxidants that drive ferroptosis can be produced, as well as substances that resist lipid oxidation. Free PUFAs have been shown to require activation and binding to membrane lipids such as phospholipids (PLs) to mediate peroxidation, and the bound products are specific lipids that cause cell death (20). The majority of biological processes require free long-chain fatty acids to be transformed into the appropriate acyl coenzyme A, and ACSL4 is a member of the enzyme family of acyl-CoA synthetase long-chain isoforms (ACSL) that catabolizes and metabolizes many PUFAs such as arachidonic acid (AA) eicosapentaenoic acid (EPA) and is a specific metabolic isoenzyme (21). Using two separate techniques, one study established ACSL4 as a significant predictor of ferroptosis susceptibility and discovered that ACSL4 was the enzyme in the ACSL enzyme family that was specifically connected to ferroptosis, independent of the other ACSL enzymes (22). Resistance to sorafenib, a medication that induces ferroptosis, has been a significant obstacle in the treatment of individuals with hepatocellular carcinoma. The main role is played by microRNAs (miRNAs), and the regulatory mechanism involves miR-23a-3p. Proteomics analysis revealed that miR-23a-3p inhibitors could prevent ACSL4 overexpression and reduce sorafenib-induced lipid peroxidation and cellular iron accumulation, suggesting that miR-23a-3p can affect ferroptosis by regulating ACSL4 to affect sorafenib resistance in hepatocellular carcinoma (23). Previous research has demonstrated that ferroptosis is invariably linked to the development of gliomas, that inhibiting ferroptosis increases glioma metastasis and proliferation, and that glioma growth may be correlated with a decline in ferroptosis. ACSL4 was shown to be significantly downregulated in gliomas, and low expression of ACSL4 reduced ferroptosis sensitivity and increased glioma cell viability (24). Cdh16Cre-ACSL4F/F mice were obtained after knocking out the ACSL4 gene in the renal tubules of mice with acute renal failure, and ACSL4 knockout mice had a significant reductions in ferroptosis and some improvements in the pathological damage associated with renal failure. This indicates that ACSL4, a key factor of ferroptosis, is a viable target for acute kidney damage (25). Numerous research projects have demonstrated that ACSL4 inactivation is a crucial mechanism for preventing ferroptosis in various settings. Overexpression of ACSL4 in cancer cells can result in ferroptosis sensitivity and cell death, which has important therapeutic implications for the treatment of several illnesses.

The enzyme lysophosphatidylcholine acyltransferase 3 (LPCAT3) is essential for the reacylation stage of phospholipid remodeling and can facilitate the esterification of unsaturated fatty acids that are broken down by ACSL4 (22). It also plays a central role in ferroptosis and is one of the genes that drive ferroptosis. Free PUFAs have been shown to require activation and binding to membrane lipids such as PLs to mediate peroxidation, and the bound products are the specific lipids that cause cell death (26). By integrating PUFAs into cellular phospholipids (particularly phosphatidylethanolamine) to create the specific lipids that cause ferroptosis, ACSL4 and LPCAT3 play a significant part in mediating ferroptosis (27). Inhibiting LPCAT3 can make cancer cells less sensitive to ferroptosis, and experimenters exposed kidney cancer cell lines treated with LPCAT3 inhibitors to certain concentrations of ferroptosis inducers and found that the treated cells were insensitive to ferroptosis, while the levels of arachidonic acid phospholipids decreased significantly, indicating that inhibiting LPCAT3 decreased the synthesis of specific lipids that trigger ferroptosis, and the cells were much less likely to undergo ferroptosis, confirming the important role of LPCAT3 in ferroptosis (28). LPCAT3 and ACSL4 play critical roles. in controlling the lipid peroxidation process. Managing these factors can effectively inhibit ferroptosis to treat various disorders.

#### 2.2.2 Reactive oxygen

Cancer cells need high levels of ROS to divide, and they create high levels of ROS through mitochondrial oxidative metabolism. Antioxidants are needed to remove ROS and maintain oxidative stress equilibrium. GSH is the main antioxidant in the antioxidant system, and can prevent oxidant-induced oxidative stress (29). The primary enzyme for reductive peroxidation is GPX4, which converts reduced GSH into oxidized GSH. This factor is also linked to ferroptosis and is crucial for the prevention of peroxidation. Cells are vulnerable to ferroptosis when GPX4 is inhibited or destroyed, leading to cell death. An experimental study demonstrated that dihydroartemisinin could cause ferroptosis in glioblastoma, which reduced the amount of GPX4 and caused tumor cells to die (30). In addition, a peroxide called FINO_2_ can cause ferroptosis, and some investigations have found that FINO_2_ indirectly impairs the GPX4 enzyme function. By using liquid chromatography–mass spectrometry to analyse GPX4 in the lysates of cells treated with carriers or inducers of iron shedding, researchers discovered that FINO2 reduced GPX4 activity in the lysate to a similar extent and that FINO_2_ decreased GPX4 activity, indicating that FINO_2_ is a direct inhibitor of GPX4 or that FINO_2_ can deplete GPX4 protein (31). Legumin plays a crucial part in the management of acute kidney injury. It has been discovered that legumin can mediate the development of acute kidney damage by controlling the degradation of GPX4, legumin and GPX4 can interact to promote autophagy, legumin deficiency can ameliorate acute kidney injury in mice, and legumin is a key target of acute kidney injury (32). If GPX4 activity is compromised, it can directly result in ferroptosis. GPX4 may be a crucial target for controlling ferroptosis because it is a critical factor in determining whether ferroptosis takes place.

A second antagonistic mechanism of ferroptosis involves coenzyme Q_10_ (CoQ_10_), which prevents lipid oxidation during ferroptosis. Apoptosis-inducing factor mitochondrial 2 (AIFM2) is also referred to as ferroptosis inhibitory protein (FSP1, is necessary for CoQ_10_ synthesis). Numerous cancer cell lines express FSP1, a powerful death inhibitor, and ferroptosis is positively correlated with this factor. The FSP1/CoQ_10_ system and the common GPX4 glutathione pathway act in parallel. Even if GPX4 is functional, deletion of FSP1 increases phospholipid oxidation, and FSP1 inhibits ferroptosis by preventing lipid peroxidation through a mechanism that differs from the glutathione-dependent protective pathway (33). Kelch-like ECH-associated protein 1 (KEAP1) in lung cancer typically mutates or is inactivated. It has been discovered that inhibiting CoQ_10_ synthesis can overcome the radiation resistance of lung cancer cells or KEAP1-deficient or mutated tumors, inhibiting FSP1 makes KEAP1-deficient lung cancer cells radiosensitive by inducing ferroptosis. The KEAP1-Nrf2 pathway’s primary downstream effector is FSP1/CoQ10, which is found in KEAP1-mutant lung cancer and identified as a critical therapeutic target (34). The stability of the FSP1/CoQ_10_ system also influences how sensitive cells are to ferroptosis, making it a crucial monomers of ferroptosis research.

Another antioxidant system that contributes to ferroptosis is the GTP cyclic hydrolase-1 (GCH1)-tetrahydrobiopterin (BH4) pathway. Overexpression of GCH1 restores BH4 levels and reduces radiation-induced ROS generation, while deletion of BH4 causes increased ROS levels (35). Research has shown that BH4 controls how sensitive colorectal cancer (CRC) cells are to ferroptosis caused by erastin. Resistance to erastin and RSL3 resistance, two classic inducers of ferroptosis, is observed in many CRC cell lines. The initial rate-limiting enzyme of BH4 is GCH1. Inhibiting GCH1 through genetic or pharmaceutical means reduces BH4 levels and mediates erastin-induced cell death. GCH1 blockers combined with erastin may be a potential treatment approach for CRC by increasing erastin-induced ferroptosis through activation of the ferritin phagosome (36). Considerable CoQ_10_ enrichment is observed in cells overexpressing GCH1, which also increases GCH1 sensitivity and controls ferroptosis (37). Consequently, by preventing lipid peroxidation, the GCH1-BH4 pathway controls ferroptosis.

#### 2.2.3 Iron-driven lipid peroxidation and iron metabolism

Maintaining iron homeostasis is a requirement for regular cellular activities because the body needs iron, a vital trace element. When there is excess Fe^2+^ in cellular storage when iron metabolism is disrupted, the Fenton reaction occurs. As a result of the Fenton reaction, many hydroxyl radicals enter cells, where they cause lipid peroxidation and the accumulation of ROS. Additionally, the catalytic subunit of lipoxygenase (LOX), which catalyses the lipid peroxidation reaction and results in ferroptosis and cell death, is crucially dependent on Fe^2+^. The well-known cellular iron regulators ferritin (TF) and transferrin receptor 1 (TFR1) transform Fe^2+^ into Fe^3+^ to stop the Fenton reaction. Extracellularly, TF transports two Fe^3+^ to TFR1 and intracellularly forms the TF-(Fe^3+^)^2^-TFR1 compound in the lysosome, where Fe^3+^ is degraded to Fe^2+^ and stored in ferritin through the action of iron reductase (STEAP3). By regulating the levels of the storage protein ferritin through ferritin phagocytosis, iron abundance and the sensitivity to ferroptosis are regulated. Serum anti-transferrin antibodies and TF depletion can prevent ferroptosis in mouse embryonic cells (38). Numerous cancer cells highly express TfR1, including those found in breast, lung, and colorectal malignancies, according to studies. In glioblastoma, increased expression of TF and TfR1 promotes iron uptake, cell cycle progression and cancer cell stemness. Antibodies against TfR1 can effectively inhibit cancer cell proliferation (39). The cell stores iron in an erratic iron pool. The majority of cellular iron is transferred to the mitochondria for use in the synthesis of haem or iron–sulfur (Fe-S) clusters, which can then be exported from the mitochondria to the cytoplasm via the mitochondrial receptors FLVCR haem transporter 1 (FLVCR1) and ATP-binding cassette subfamily B member 7 (ABCB7), respectively. An iron-containing mitochondrial outer membrane protein called CDGSH iron–sulfur domain 1 limits mitochondrial iron intake and prevents ferroptosis (40).

**Figure 1:**
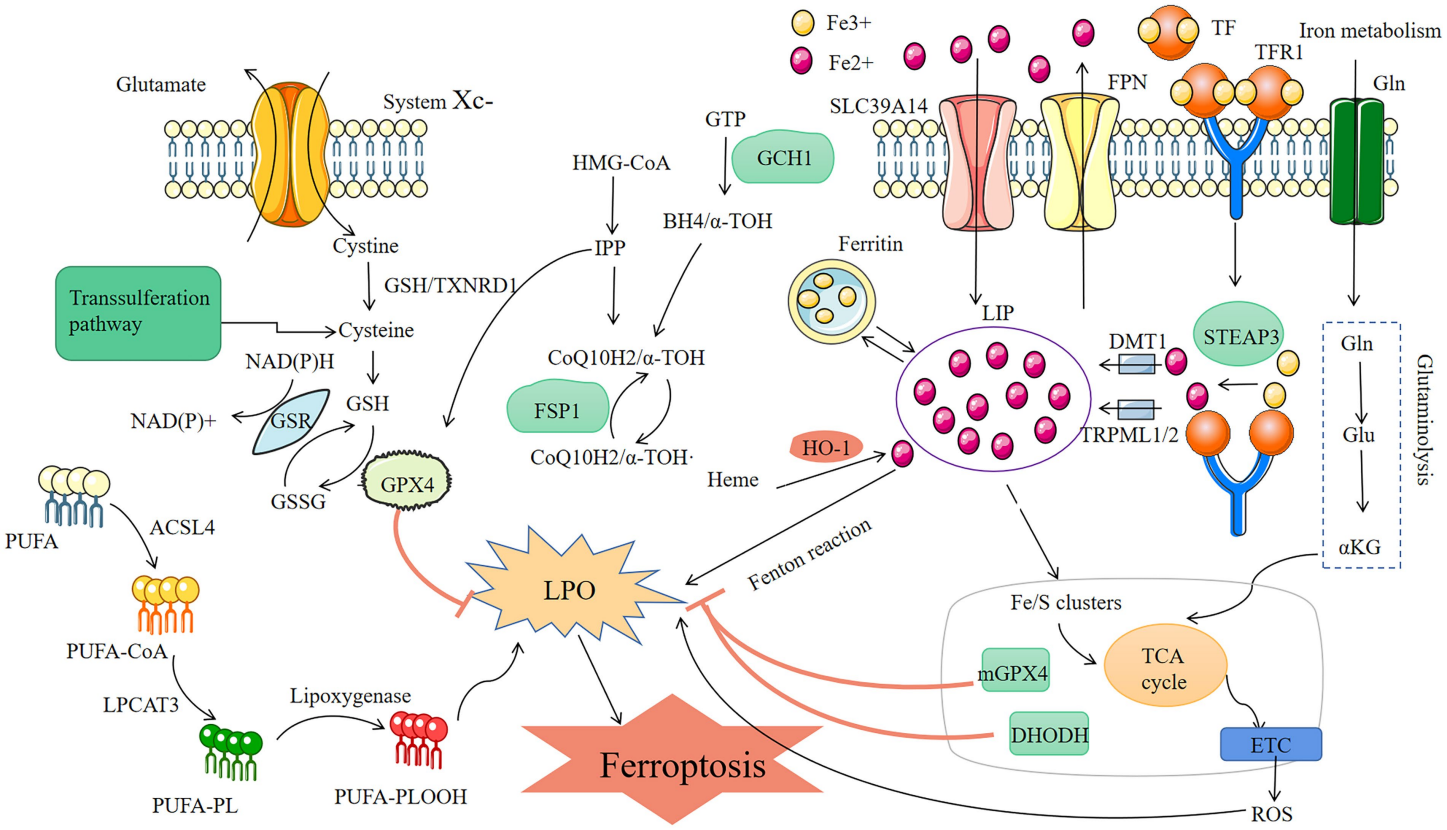
Mechanisms of ferroptosis three metabolic pathways of ferroptosis: lipid metabolism, conversion of PUFA into lipid peroxidation-causing substances by ACSL4 and LPCAT3 leads to cellular ferroptosis; reactive oxygen, GPX4 prevents oxidative stress by reducing GSH and thus inhibits ferroptosis; iron metabolism, the Fenton reaction, which develops in the presence of too much Fe2+ and results in the buildup of reactive oxygen species and lipid peroxidation-induced ferroptosis, TFR1 and TF are the key to control Fe2+.

Hepcidin, a peptide produced and secreted by the liver, has a detrimental effect on the control of iron homeostasis (41). Iron can only be transported from cells to the blood during iron metabolism by membrane iron transporter protein 1 (FPN1). Hepcidin interacts with FPN1 to enhance internalization and degradation, indirectly controlling iron homeostasis. When the body has too much iron, the liver increases hepcidin gene expression to produce and secrete more hepcidin (42). As a result, FPN1 is broken down more quickly, the export of iron for transport to the blood is inhibited, and less iron is transported to the blood by small intestinal epithelial cells and macrophages. When the body is deficient in iron, these processes undergo the opposite changes, thus maintaining iron homeostasis. Hepcidin inhibitors, hepcidin inducers, and iron transporter inhibitors (ebselen) were administered to model rats with subarachnoid haemorrhage (SAH) after induction. The findings demonstrated that ebselen inhibited the iron transporter DMT1 to prevent iron accumulation and lipid peroxidation in SAH rats, reducing ferroptosis and early brain injury (EBI), showed that heparin decreased the expression of hepcidin and DMT1, elevated FPN1 levels, and had comparable inhibitory effects on ferroptosis and EBI as ebselen. In addition, ebselen restored the effects on iron failure, while hepcidin inducers enhanced the expression of hepcidin and DMT1, decreased FPN1, and aggravated iron failure and EBI. Hepcidin modulates iron metabolism and facilitates ferroptosis in EBI rats following SAH by activating the DMT1 signalling pathway (43). These are the primary ferroptosis-regulating systems are shown in Figure 1. In addition, there are other antioxidant systems involved in the regulation of ferroptosis, but the specific mechanisms and roles need to be further clarified.

## 3 Ferroptosis and NAFLD/NASH

**Figure 2:**
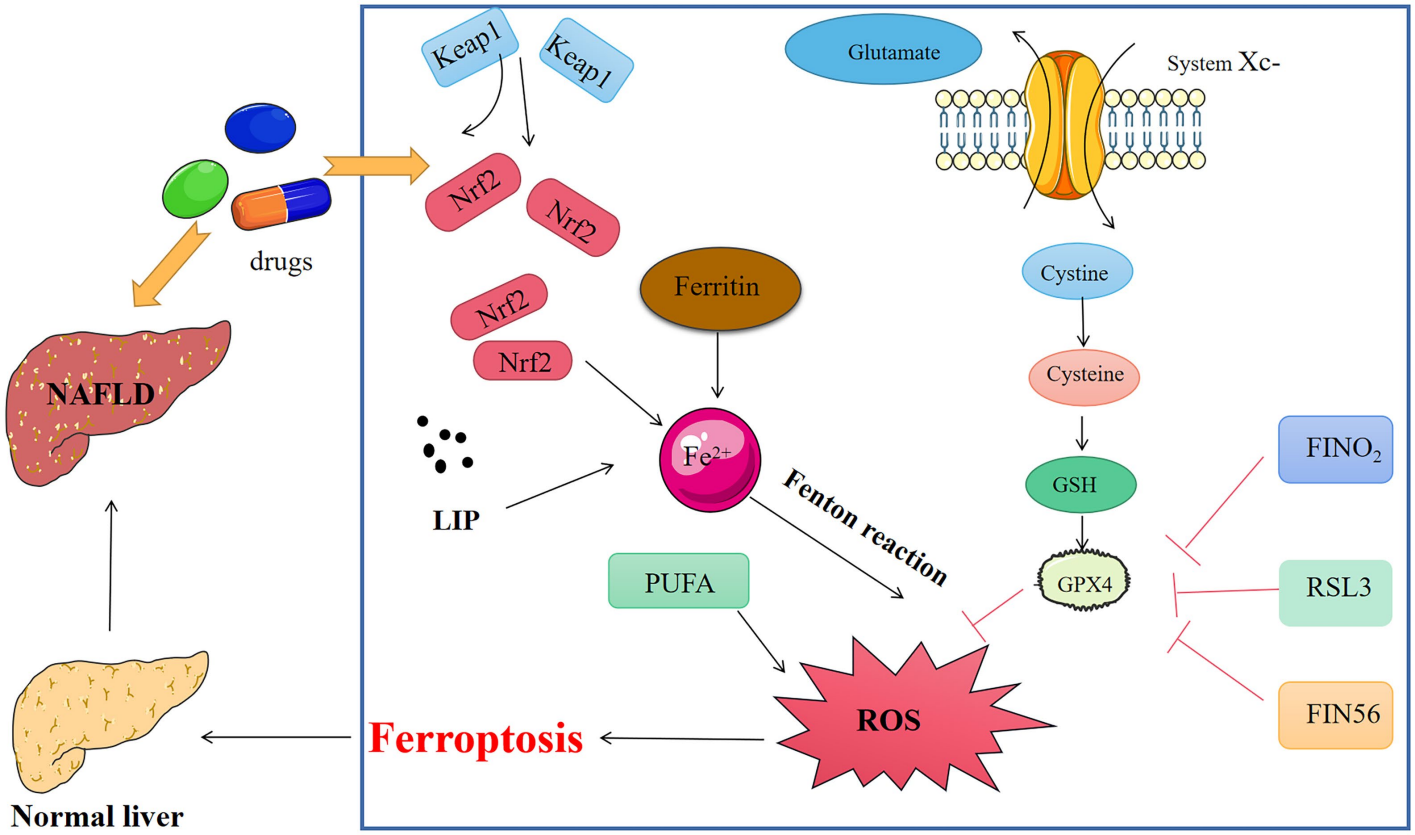
Medicine interferes with key chemicals, genes, and metabolic networks for ferroptosis in NAFLD.

Due to the liver’s sensitivity to oxidative damage and the fact that excess iron and oxidative stress are the two primary initiators of liver injury and disease progression in the majority of liver illnesses, ferroptosis has recently received much attention recently in the field of liver diseases (44). The primary organ for storing iron and metabolizing lipids is the liver. When the liver is impaired, the levels of iron and lipid ROS are significantly elevated (45). Patients with NAFLD often have increased serum ferritin levels, which are linked to intrahepatic iron accumulation;, iron levels are markedly elevated in the liver when the liver is dysfunctional. NAFLD is mostly caused by lipid peroxidation caused by iron (46). Additionally, insulin resistance and obesity, both of which are common in NAFLD patients, are linked to iron deficiency. The connection between ferroptosis and NAFLD/NASH is becoming increasingly clear: increased levels of lipid peroxide and upregulated expression of essential ferroptosis proteins occur in NAFLD livers, and that ferroptosis inhibitors efficiently prevent ferroptosis and mitigate NAFLD. Ferroptosis is now recognized as a critical target for curing liver illnesses. According to a pertinent bioinformatic study, NAFLD livers have lower levels of selenium than healthy livers, and selenium is a crucial part of the ferroptosis-related protein GPX4. Additionally, statistical analysis, showed that almost one-third of patients had elevated liver iron levels, which may be the result of aberrant iron metabolism that contributes to the onset of disease (47). Much emphasis is also being paid to the investigation of iron-associated mortality in NAFLD/NASH. Determining the fundamental regulatory mechanisms and understanding how ferroptosis affects NAFLD/NASH are crucial issues (Figure 2).

### 3.1 Lipid peroxidation causes ferroptosis in NAFLD/NASH

Lipid peroxidation is a significant contributor to NAFLD/NASH. Lipid peroxidation products damage the majority of hepatocytes in the liver as NAFLD progresses. Hepatocytes are damaged by lipid peroxidation, and these processes also deplete intracellular antioxidants, which causes a lack of GSH and vitamin E and feeds a vicious cycle of oxidative imbalance (48). Animals treated with RSL-3 (a ferroptosis inducer) and fed a methionine/choline-deficient diet (MCD) for 10 days after developing NASH exhibited decreased hepatic GPX4 expression. Treatment of MCD-fed mice with the GPX4 activator sodium selenite increased hepatic GPX4 expression and decreased the severity of NASH, indicating that GPX4 levels were impaired in NASH patients and therefore caused lipid peroxidation-induced ferroptosis (48). In experiments using ferroptosis inhibition and necrosis inhibition, necrosis in liver cells was not significantly improved in mice treated with necrosis inhibitors; in contrast, ferroptosis inhibition almost entirely prevented necrotic death in the hepatocytes of mice feds choline-deficient, methionine-supplemented (CDE) diet. This anomaly was explained by the discovery that necrosis occurred before the commencement of apoptosis. Additionally, there was a rise in the quantity of ferroptosis-related oxidized phosphatidylethanolamine. These results indicate that hepatic ferroptosis is a key player in steatohepatitis-associated inflammation (3). Quercetin, which reduces MtROS-mediated lipid droplet production in steatotic L-02 cells, can reduce lipid ROS levels and ferroptosis and has been shown to reverse hepatic lipid peroxidation, lipid accumulation, and ferroptosis caused by a HFD (49). These results indicate that ferroptosis induced by lipid peroxidation plays a crucial role in the development of NAFLD.

### 3.2 The Nrf2 pathway causes ferroptosis in NAFLD/NASH

Ferroptosis is correlated with nuclear factor E2-related factor 2 (Nrf2) activity. Ferroptosis is sensitive to Nrf2 gene inactivation, repression, or knockdown; however, Nrf2 signal pathway activation can reduce ferroptosis susceptibility and alleviate NAFLD/NASH. It was discovered that dehydrofirmaic acid (DA) affects ferroptosis in NAFLD mice and that DA could intensify the action of Nrf2 antioxidant-responsive element luciferase activity by binding to Keap1. This increases the expression of haem oxygenase-1 (HO-1), GSH, and GPX4, eliminates the accumulation of ROS, reduces the levels of lipid peroxide and suppresses ferroptosis (50). Similarly, Ginkgolide B (GB), a key monomers of *Ginkgo biloba* extract, reduced NAFLD in mice fed a high-fat diet, and the WB results revealed the expression levels of ferroptosis-related proteins such as Nrf2, GPX4, HO-1, TFR1, and ferritin heavy chain-1 (FTH1) *in vitro* and *in vivo*. Nrf2 interference following, GB therapy dramatically increased Nrf2 expression, suggesting that GB can be used to treat ferroptosis induced by lipid accumulation and oxidative stress in response to NAFLD via the Nrf2 signalling pathway. It was also discovered that TFR1 was upregulated, FTH1 was downregulated, and lipid peroxidation and Nrf2 activity were inhibited (51). HFD-induced downregulation of Nrf2 requires ATG7. The capacity of cells to undergo ferroptosis, which is not considerably worsened by HFD, is enhanced in ATG7-knockout mice, and lower levels of Nrf2 may be seen in these animals. This suggests that the severity of NAFLD in patients can be determined by the functional status of ATG7 (52). Nrf2, which is a critical ferroptosis gene, is intimately associated with the incidence of many diseases. An in-depth examination of the connection between Nrf2 and ferroptosis can provide precise guidelines for the treatment of diseases.

### 3.3 Iron accumulation causes ferroptosis in NAFLD/NASH

Iron has two distinct valence states, and because it participates in intracellular redox reactions in living organisms, it produces oxidative radicals. ROS are produced when there is iron overload, which causes oxidative stress (53). The rate of liver-derived transferrin synthesis is inversely associated with the amount of intracellular iron. To determine iron metabolism, transferrin measurement is as crucial as total serum iron binding capacity. Moreover, transferrin can be used as a sign of hepatocyte injury (54). Statistically, it has been found that when NAFLD occurs in advanced stages, patient serum ferritin levels are high. High levels of iron accumulation can cause severe lipid peroxidation thus triggering ferroptosis and leading to cell death. Iron accumulation is one of the manifestations of NAFLD, and iron overload in NAFLD can cause iron sagging in mice and patients and aggravate NAFLD (55). In model of NAFLD, a heavy iron diet exacerbates oxidative stress and inflammatory reactions and hastens the development of NASH (8). Additionally, primary haemochromatosis worsens NASH, while iron withdrawal reduces ALT levels and liver damage in these individuals (56). Hepcidin binds directly to iron transporters to stop the flow of iron into plasma, and too much iron has been linked to a number of illnesses and disorders connected to obesity. Hepcidin regulates major iron export, protects against hepatic steatosis, enhances glucose tolerance and insulin sensitivity, and reduces obesity caused by high-fat diets. Hepcidin inhibitor Tmprss6 deficiency was shown to increase hepatic lipolysis and stop the development of hepatic steatosis in mice (57). Prominin2, a pentapeptide protein linked to the control of lipid homeostasis, is induced when ferroptosis occurs in cells. This protein promotes the synthesis of transferrin, which reduces intracellular iron accumulation and renders cells less sensitive to ferroptosis. It also serves as a new pathway to combat the iron accumulation that results in ferroptosis (58). Previous studies have shown that iron overload can adversely affect insulin secretion by pancreatic β-cells and can interfere with insulin receptor expression, leading to an increase in IR. Pancreatic β-cells are highly sensitive to iron ion levels and can express hepcidin to alleviate iron overload. In addition, excessive iron accumulation induces oxidative stress and mitochondrial damage, which further impairs pancreatic β-cell function (59). Consequently, maintaining proper iron homeostasis can support cellular health and reduce the risk of illness. The amount of iron in cells has a significant impact on normal physiological processes.

## 4 TCM and monomers regulates ferroptosis to inhibit nonalcoholic fatty liver disease

Based on its potent antioxidant effects, TCM will be used more often in the clinical treatment of many diseases, and in the recent past, research on the role of TCM in ferroptosis and the management of disease has attracted much interest (60). *Pueraria lobata* (Willd.) Ohwi. improves the ability of liver cells to regenerate, returns liver function to normal, encourages bile production, and stops fat accumulation in the liver. It has been discovered that the active monomers is Puerarin, which can protect neurons by suppressing the production of ROS while controlling the quantity of iron to prevent iron overload and subsequent ferroptosis. In mice with Alzheimer’s disease, puerarin can improve learning and cognitive impairment by preventing iron overload in the cerebral cortex (61). A major active alkaloid monomers known as leonurine has been isolated and purified from *Leonurus japonicus* Houtt., and it can activate the antioxidant transcription factor Nrf2 to inhibit ferroptosis and protect against liver and kidney damage. The Nrf2/HO-1 signalling pathway is blocked by this, astragalus polysaccharides, which protect intestinal epithelial cells (IECs) from iron-induced mortality and reduce lesions in a mouse model of experimental colitis (62). Ferroptosis and stroke have been linked in several studies. During pathological stroke injury, there is an increase in iron ion accumulation and iron-dependent lipid peroxidation, and the use of ferroptosis inhibitors dramatically reduces subsequent brain tissue damage following stroke. It is clear that TCM is effective in the treatment of stroke. To prevent iron ion accumulation and lipid peroxidation damage in brain tissue, the aromatic plant volatile oil carvacrol has been shown to up regulate GPX and FPN1 expression and GSH while downregulating TFR1 expression, this prevented ferroptosis in neurons. Saffron yellow and galangin, two TCM extracts, can reduce stroke-induced brain tissue damage by blocking the ferroptosis pathway (63). The pathogenesis of NAFLD, the identification of therapeutic targets, and the advancement of drug development have all made steady progress, but there is still no specific therapeutic agent for the treatment of NAFLD/NASH, and no medication has yet been approved for use in the condition (64). Thus, regulation of ferroptosis by TCM may become a means of treating NAFLD (Table 1).

**Table 1:** Therapeutic effects of TCM on regulating ferroptosis on NAFLD.

TCM	Function	Stage	References
Ginkgolide B	Activates Nrf2, leading to reduced lipid peroxidation	Steatosis and NASH	Yang et al. (51)
Epigallocatechin gallate	Prevents GSH consumption, GPx4 inactivation and lipid peroxidation	NASH	Kose et al. (78), Ding et al. (79), and Yang et al. (80)
Quercetin	Targets mitochondrial ROS-mediated ferroptosis	Steatosis and NASH	Jiang et al. (49), Yin et al. (66), and Qin et al. (68)
Chaihu Shugan powder	Targets mitochondrial ROS-mediated ferroptosis	Steatosis and NASH	Wang et al. (87), and Nie et al. (88)
Danlou tablets	Increases Fe^2+^ accumulation, promotes the ubiquitination of IREB2 and supresses the expression of FTH1	Steatosis and NASH	Xin et al. (91) and Erwen et al. (92)

### 4.1 Monomers

**Figure 3:**
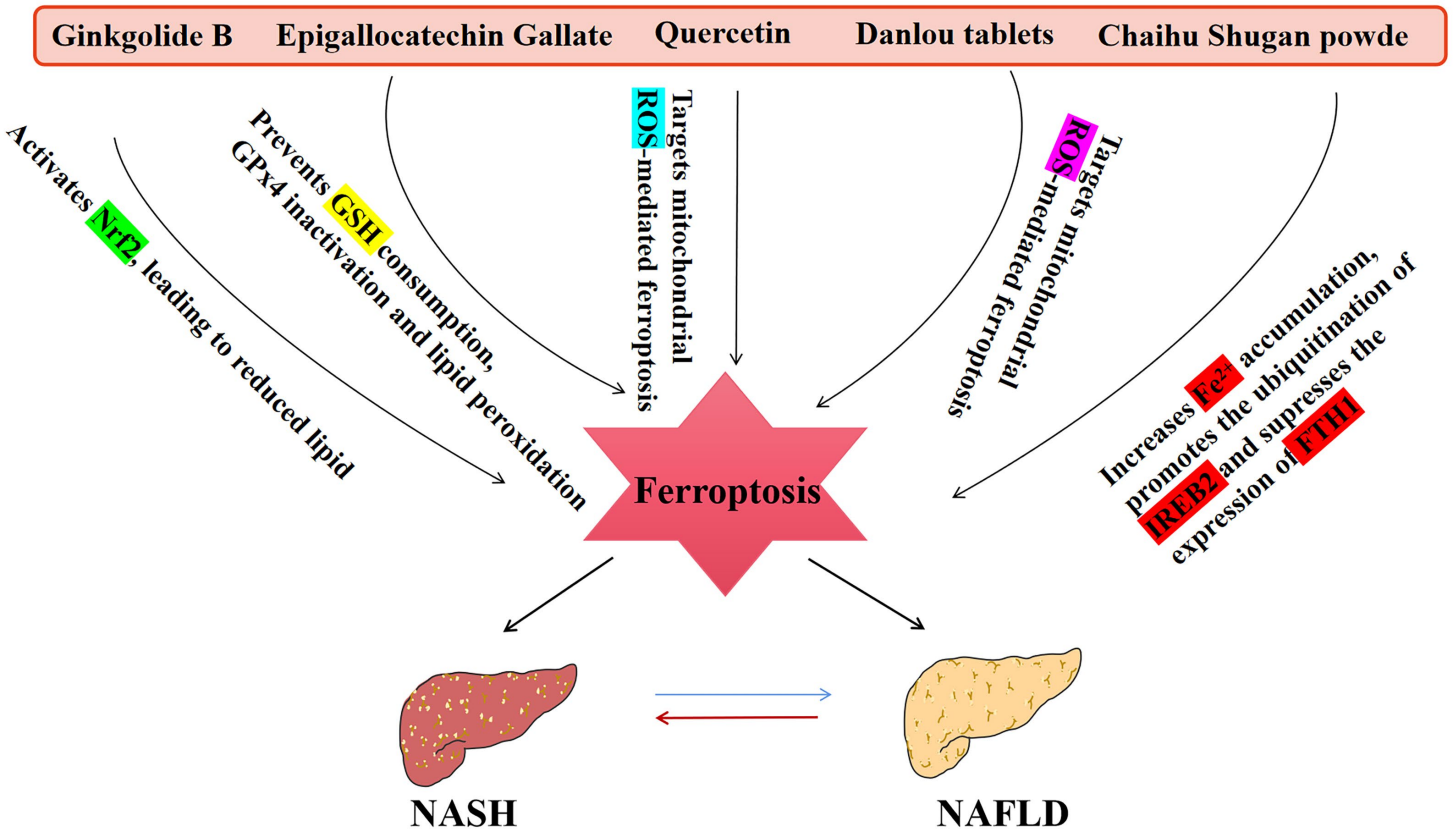
An overview of TCM that affect the NAFLD disease spectrum. NASH, non-alcoholic steatohepatitis.

Dehydrofirmanoic acid (DA) and ginkgolide B (GB), which were mentioned earlier, can be involved in the development of NAFLD/NASH by affecting the Nrf2 pathway to cause ferroptosis, and some herbal monomers can also have therapeutic effects on the disease by regulating ferroptosis (Figure 3).

#### 4.1.1 Quercetin

Quercetin is a flavonoid metabolite with excellent antioxidant and anti-inflammatory properties that can modulate multiple pathways such as Nrf2, antioxidant enzymes, and different cytokines (including IL-6, IL-10, and TNF-α) (65). Thus, quercetin can control iron metabolism in diseased cells and has good iron chelating properties. Quercetin prevents lipid peroxidation and iron chelation by scavenging oxygen free radicals. Quercetin has a unique chemical structure that allows it to bind unstable cytoplasmic iron, delay the initiation of the Fenton reaction, and simultaneously export intracellular iron. The quantity of reactive oxygen species that iron creates in cells can be reduced by quercetin, which indicates that it does so by directly chelating iron to prevent it from taking part in redox processes and reducing the vulnerability of cells to ferroptosis (66). It has been established that eating foods high in quercetin can help prevent and cure NAFLD, and one study using HFD-fed rats treated with quercetin and other medications showed that quercetin-treated rats had a 39% decrease in hepatic TG and a 1.5-fold increase in VLDL (67). Using HFD-induced male SD rats, a cell model of primary hepatocytes cocultured with Kupffer cells stimulated by lipopolysaccharide/free fatty acid (LPS/FFA) shown that quercetin significantly reduced lipid accumulation, inflammation, and oxidative damage, and another study demonstrated that quercetin regulated lipid metabolism by activating AMP-activated protein kinase (AMPK), which reduced hepatic fat accumulation, inflammation, and oxidative stress, thereby alleviating NAFLD (68). Detection of lipid peroxidation, steatosis, and ferroptosis in the liver in C57BL/6 J mice fed a normal diet (ND), a high-fat diet (HFD), and a HFD plus quercetin for 12 weeks revealed that quercetin improved the lipid peroxidation and iron overload in liver cells induced by a high-fat diet. Additionally, *in vitro* experiments with quercetin-treated L-20 cells showed attenuated steatosis and decreased lipid ROS concentrations and iron overload (49). In addition to research on quercetin in NAFLD, one study showed that quercetin had a similar impact on hepatocellular carcinoma cells by promoting lysosomal stimulation mediated by the transcription factor TFEB, which promoted ferritin breakdown and ultimately led to ferroptosis (69). Quercetin has several benefits as a natural ferroptosis inhibitor in the treatment of NAFLD, and it is important to fully understand the mechanism by which it controls ferroptosis.

#### 4.1.2 Rutin

Evidence has shown that rutin, a glycoside derivative of quercetin, can trigger the death of tumor cells and that rutin-containing anticancer medications can also have neuroprotective effects (70). Rutin can reduce the amount of cellular reactive oxygen species and promote benign alterations in the antioxidant defence system in hepatocellular carcinoma cells, avoiding or postponing circumstances that favor cellular oxidative stress (71). Rutin dramatically decreased weight gain, markers of liver injury (AST and ALT), steatosis, and hypertrophy in obese mice fed a HFD. Rutin reduced NAFLD in rats fed an HFD, and there were improvements in blood levels of total cholesterol, LDLc, and triglycerides that were equivalent to those of simvastatin (72). Trichostatin, a trihydroxyethylated derivative of rutin, increases the production of the nicotinamide phosphotransferase (NAMPT) protein, which dramatically decreases oxidative stress-mediated NAD+ depletion and decreases polypolymerase-1 protein (PARP1) expression and activity, and it effectively prevents high-cholesterol diet-induced obesity in mice; trichostatin promotes sirt1-mediated AMPK activation, which has beneficial effects on HFD-induced hepatic lipid homeostasis in NAFLD (73). Plasma glutathione peroxidase activity is reduced, malondialdehyde levels are increased, and superoxide generation if increased by erythrocytes, more severe oxidative stress is experienced by rats fed a high-fat diet for 16 weeks. Similarly, a small amount of rutin added to a high-fat diet can moderate the effects of diet-induced metabolic syndrome, NASH, and cardiovascular problems by restoring normal hepatic marker expression, reducing liver and cardiac inflammation and oxidative stress, and altering lipid metabolism to some extent (74). *In vitro* and *in vivo* studies have shown that rutin possesses antioxidant and iron-chelating effects. Rutin treatment was performed on an iron overload genetic mouse model, and WB analysis of liver ferritin levels and RT-PCR analysis of liver iron homeostasis genes showed that rutin therapy dramatically reduced serum transferrin saturation and hepatic ferritin expression. Furthermore, an increase in serum unsaturated iron binding capacity and decrease in liver and serum iron levels was observed (75). Due to rutin’s ability to chelate iron, it may be able to prevent NAFLD and be useful in treating disorders where iron buildup occurs (76). However, further research is needed to determine the precise structural and pharmacodynamic mechanism of rutin’s therapeutic effects.

#### 4.1.3 Epigallocatechin gallate

As the main monomers of green tea polyphenols, epigallocatechin gallate (EGCG) has been shown to be helpful in metabolic diseases (77). In addition to functioning as a natural iron chelator, EGCG substantially inhibits cellular GSH depletion, GPX4 degradation, and lipid peroxidation levels in pancreatic cells (78). Previous studies have confirmed that EGCG, as a polyphenol component of green tea, may play a protective role in liver lipotoxicity by inhibiting mitochondrial reactive oxygen species mediated liver ferroptosis, and is a potential inhibitor of ferroptosis (79, 80). Due to its antioxidant and lipid metabolism modulating effects EGCG may be useful in the treatment of NAFLD, and it has been shown to reduce the effects of proinflammatory cytokines and oxidative stress molecules (81). EGCG could lower body weight and liver weight in mice with NASH induced by a methionine and choline deficient (MCD) diet, which may be explained by the fact that EGCG reduces liver fibrosis and regulates oxidative stress and inflammatory responses (82). Rats fed a high-fat diet received intraperitoneal injections of EGCG. Serum and liver tissues were examined, and EGCG decreased the expression of key pathological oxidants (such as nitrotyrosine) and proinflammatory markers (such as NOS, COX-2, and TNFα) and inhibited TGF/SMAD, PI3K/Akt/FoxO1, and NF-κB pathway activity (83). ACSBG, a long-chain fatty acid coenzyme necessary for fatty acid metabolism and the ferroptosis pathway, was highly expressed in the liver cells of NASH mice treated by gavage or intraperitoneal injection of EGCG. This treatment improved lipid accumulation and ferroptosis, thus preventing the development of NASH (84). The regulatory effect of EGCG on NAFLD/NASH is closely related to ferroptosis, which remains to be verified in depth.

### 4.2 Compounds

#### 4.2.1 Chaihu Shugan powder

Chaihu Shugan powder (CSP) is a Chinese medicine that is used to dredge the liver and regulate Qi, energize blood, and reduce pain (85), and it is a general remedy for NAFLD. CSP is composed of Radix Bupleuri, *Citrus Reticulata*, Chuanxiong Rhizoma, Paeoniae Radix Alba, Aurantii Fructus, Cyperi Rhizoma and Radix Glycyrrhizae. In this compound, Radix Bupleuri is good at draining the liver and relieving depression. The combination of the two botanical drugs helps Radix Bupleuri relieve the stagnation of the liver meridian and increases the promotion of the flow of Qi and blood to relieve pain. *Citrus Reticulata* and Aurantii Fructus regulate Qi and promote stagnation, while Paeoniae Radix Alba and Radix Glycyrrhizae nourish the blood and soften the liver to relieve pain. Radix Glycyrrhizae harmonizes all the medicines and is the botanical drug of choice (86). The combination of these the botanical drugs can promote liver draining and Qi, invigorating the blood and relieving pain. Quercetin, kaempferol, naringenin, isorhamnetin, and flavopiridol in the formula were shown to be closely related to the genes PPAR, FXR, and PPAR in NAFLD (87). Subsequent research revealed that mice with NAFLD shown improvements in body weight, liver histopathology and serum and liver lipids after treatment with CSP (88).

#### 4.2.2 Danlou tablets

Danlou tablet (DLP) is mainly composed of 10 Chinese botanical drugs, including *Ligusticum chuanxiong* Hort., *Salvia miltiorrhiza* Bunge, *Trichosanthes kirilowii* Maxim., *Allium macrostemon* Bunge., *Pueraria lobata* (Willd.) Ohwi, *Paeonia lactiflora* Pall., *Curcuma aromatica* Salisb., *Davallia marisii* Moore ex bak., *Alisma plantago-aquatica* Linn., and *Astragalus membranaceus* (Fisch.) Bunge. In this compound, *Trichosanthes kirilowii* Maxim., *Allium macrostemon* Bunge., *Curcuma aromatica* Salisb., and *Davallia marisii* Moore ex bak. Can lower blood lipid levels and inhibit atherosclerosis. *Pueraria lobata* (Willd.) Ohwi, *Alisma plantago-aquatica* Linn., and *Astragalus membranaceus* (Fisch.) Bunge. protect vascular endothelial cells against oxidative stress, *Ligusticum chuanxiong* Hort., *Salvia miltiorrhiza* Bunge, *Paeonia lactiflora* Pall., have effects on reducing capillary permeability, improve blood flow, inhibit platelet coagulation and reduce blood viscosity (89). Modern pharmacological studies have shown that Danshen tablets can reduce blood lipid levels, improving vascular endothelial function and liver lipid deposition and treating atherosclerosis. DLP has the effect of resolving blood stasis Davallia marisii Moore ex bakand disperses nodules, benefiting Qi and promoting Yang, and modern pharmacological studies have shown that DLP can reduce blood lipids and improve liver deposition (90). The NAFLD model was created by high-fat feeding, and after 4 weeks of gavage treatment, serum and other markers in the model group were examined. Mice fed DLP had significantly decreased TC, TG, ALT, AST, and Fe^2+^ serum levels. DLP may be able to treat NAFLD by reducing oxidative stress in liver cells and controlling ferroptosis because increased levels of GPX4 and ferritin heavy chain FTH1 mRNA and protein expression can reduce the levels of the key ferroptosis-related protein iron response element binding protein 2 (IREB2) in the liver (91). The ability of Zexie decoction ability to treat NAFLD by controlling ferroptosis was confirmed when it was discovered that Zexie decoction could increase the levels of FSP1, dose-dependently upregulate Nrf2 nuclear protein expression and significantly reduce ACSL4 mRNA expression (92).There are many TCM compound formulas for the treatment of NAFLD. Due to the difficulties of conducting compound research, there have been few studies on TCM compound formulas that have therapeutic effects on ferroptosis, and more thorough study is needed.

## 5 Conclusion and perspectives

The prevalence of metabolic disorders is increasing as a result of changing lifestyles, and NAFLD has emerged as one of the major illnesses (93). As a new therapeutic target related to NAFLD, ferroptosis is of great importance. Further research has revealed a link between ferroptosis and the pathogenic processes of lipid accumulation, inflammatory infiltration, fibrosis, and mitochondrial damage in NAFLD/NASH. The progression of NAFLD may be influenced by iron excess or iron-dependent lipid peroxidation, and the primary ferroptosis regulators and antioxidant pathways may be the focus of NAFLD treatment. The most important pathway is Nrf2/GSH/GPX4, which is a key antioxidant pathway that may function as a conduit for treating NAFLD by targeting ferroptosis. TCM therapy provides certain advantages in the treatment of metabolic illnesses because of the unique benefits of TCM, which may regulate ferroptosis via numerous targets to achieve therapeutic outcomes, and many TCM extracts and monomers can act as natural inhibitors of ferroptosis. Unfortunately, there are no obvious signs that clearly illustrate the precise connection between ferroptosis and the onset of illnesses, and it is yet not clear how TCM can control ferroptosis to treat different diseases. There are also great limitations in the research on the treatment of diseases by ferroptosis in traditional Chinese medicine, especially the research task of traditional Chinese medicine compound is extremely arduous. Due to the complexity of the composition of the compound, there are many problems in the study design. By isolating and analyzing the components of TCM compound extracts, it is possible to find the specific monomers that are effective in inhibiting or promoting ferroptosis and thus treating diseases; the treatment of diseases by TCM monomers by affecting ferroptosis can be manifested by the degree of lipid peroxidation, the degree of iron accumulation, and so on, which may be a better way of observing ferroptosis. Future research should focus on the mechanism of TCM in the treatment of NAFLD and other diseases by controlling ferroptosis, and the research on which specific monomers of TCM can inhibit or promote ferroptosis should be the research focus in the field of TCM.

## Author contributions

NW: Writing – original draft, Investigation, Funding acquisition, Writing – original draft, Investigation,Funding acquisition. HQ: Writing – review & editing. QL: Writing – review & editing. WZ: Writing – review & editing. HL: Writing – review & editing. QW: Funding acquisition, Supervision, Validation, Writing – review & editing, Funding acquisition, Supervision, Validation, Writing – review & editing. JG: Funding acquisition, Resources, Supervision, Writing – review & editing, Funding acquisition, Resources, Supervision, Writing – review & editing.
