# Supplementary material for: Mechanisms of ferroptosis in nonalcoholic fatty liver disease and therapeutic effects of traditional Chinese medicine: a review
Source: Front Med (Lausanne). 2024 Mar 25;11:1356225. doi: 10.3389/fmed.2024.1356225 (PMC10999571; doi:10.3389/fmed.2024.1356225)
Supplement: Supplementary file 1 [file Data_Sheet_1.docx]

Supplementary Material

Mechanisms of ferroptosis in nonalcoholic fatty liver disease and therapeutic effects of traditional Chinese medicine: A review

Nan Wang^1^, Hanyun Que^1^, Qiulin Luo^1^, Wenxin Zheng^1^, Hong Li^1^, Qin Wang^1,2*^, Jian Gu^1^*

First Author*, Co-Author, Co-Author

*** Correspondence:** Corresponding Author:

Email:gujiancd@163.com (J.G.); jiajiawangqin@163.com (Q.W.)

##
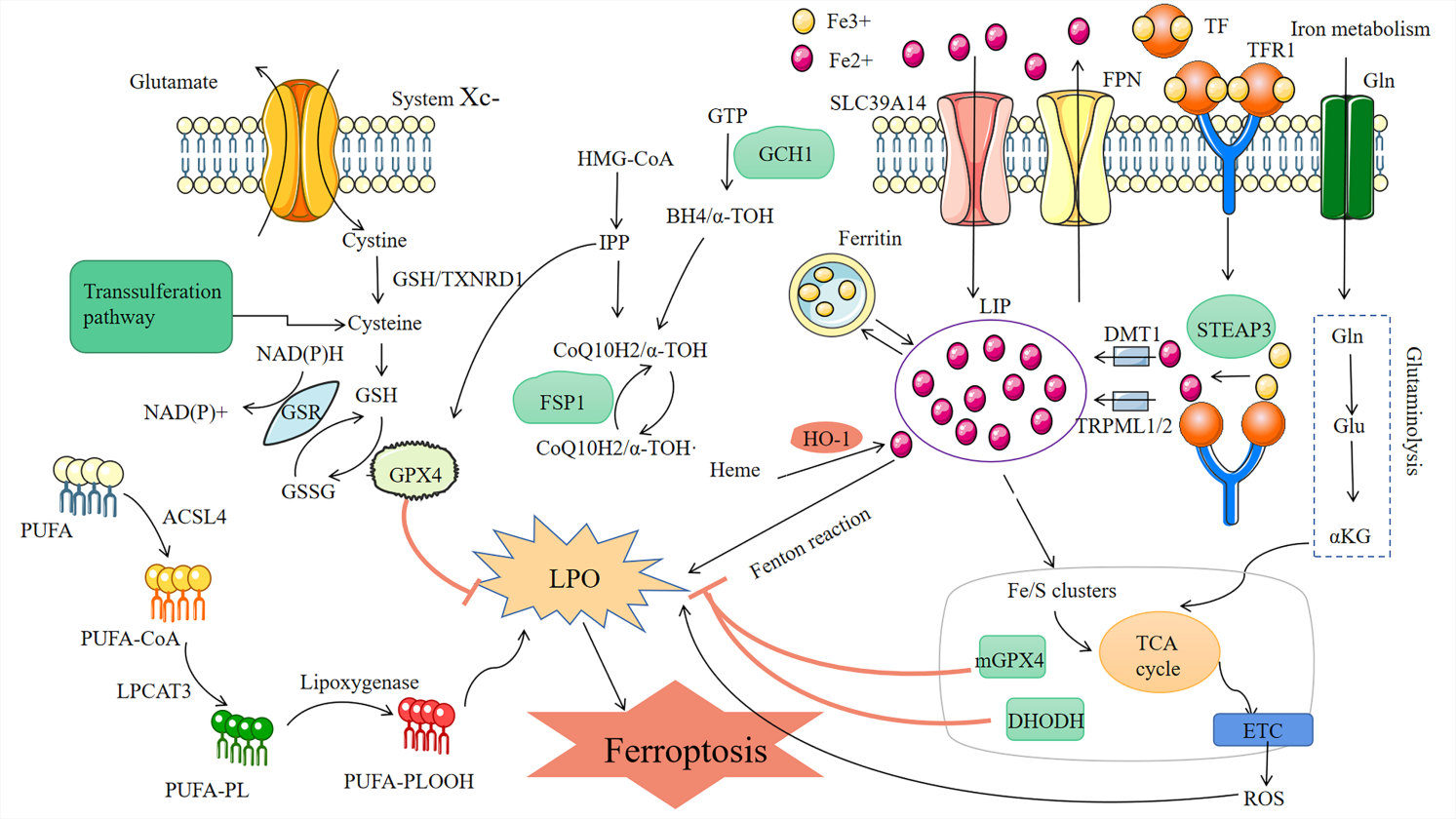
 Supplementary Figures

**Supplementary Figure 1.** Mechanisms of ferroptosis

Three metabolic pathways of ferroptosis: lipid metabolism, conversion of PUFA into lipid peroxidation-causing substances by ACSL4 and LPCAT3 leads to cellular ferroptosis; reactive oxygen, GPX4 prevents oxidative stress by reducing GSH and thus inhibits ferroptosis; iron metabolism, the Fenton reaction, which develops in the presence of too much Fe2+ and results in the buildup of reactive oxygen species and lipid peroxidation-induced ferroptosis, TFR1 and TF are the key to control Fe^2+^.

**Supplementary Figure 2.** Medicine interferes with key chemicals, genes, and metabolic networks for ferroptosis in NAFLD.


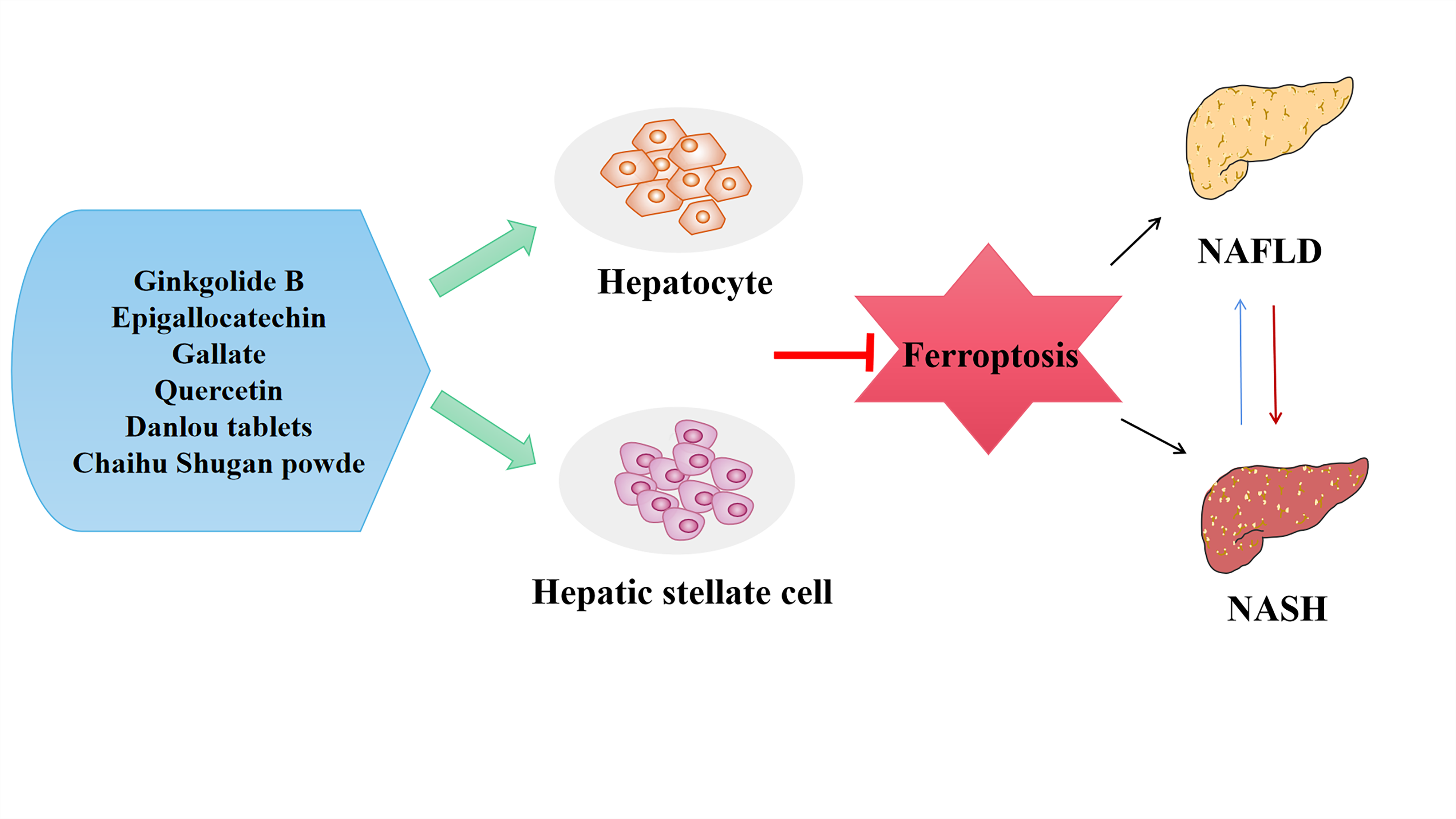


**Supplementary Figure 3.** An overview of TCM that affect the NAFLD disease spectrum. NASH, non-alcoholic steatohepatitis.
